# Topics of Public Interest

**Published:** 1913-01

**Authors:** 


### TOPICS OF PUBLIC INTEREST.

W. B. Saunders Company, medical publishers, are now established in their new building on West Washington Square—an ideal site right in the heart of Philadelphia's new publishing center.

The remarkable success of this house and the rapid growth of their business, with the increased facilities which this growth demanded, necessitated removal to large quarters. They therefore erected a seven story building, housing all their departments under one roof.

Constructed of reinforced concrete, the building is absolutely fireproof and equipped with every modern aid for the manufacture and distribution of medical books and for the comfort and convenience of their employees.

A cordial invitation is extended the profession to inspect the new plant.

Immigration and Emigration. As there is a popular idea that almost all foreigners who come to this country, except as cabin passengers as globe trotters, remain here, the following statistics may be of interest. These are condensed and approximated from figures kindly furnished by the Bureau of Immigration and Naturalization. Regarding the "Yellow Peril," the figures are as follows:

| Chinese | | | Japanese | |
|---|---|---|---|---|
| Entered | Left U. S. | | Entered | Left U. S. |
| 1263 | 3898 | Year ending June 30, 1908 | 16418 | 5323 |
| 8014 | 9565 | Year ending June 30, 1909 | 4443 | 7493 |
| 6516 | 7650 | Year ending June 30, 1910 | 4146 | 7510 |
| 5657 | 7065 | Year ending June 30, 1911 | 6490 | 8333 |
| 5491 | 6453 | Year ending June 30, 1912 | 9430 | 8080 |
| 26,941 | 34,631 | | 40,927 | 36,739 |

Without entering into details, it may be said that, in any one year, the emigration amounts to a third or even a half of the immigration. Naturalized citizens are not included in the emigraton statistics. Years ago, practically every immigrant remained as a permanent resident, if he did not become a citizen, and those that returned to the mother country were few, consisting mostly of transient visitors and of aged persons going home to spend their declining years. At present, it is a common thing to find in Europe, persons in active business life, who have spent a few years in America. Sociologists, indeed claim that in various districts, mainly agricultural or manufacturing, returned .immigrants, taking back with them comparatively small savings for America, but relatively large fortunes for Europe, and habits of enterprise, have revolutionized economic conditions so that there has been established an opportunity for development that successfully competes with the former call of the western world.

Of late years, the plurality, sometimes the majority of immigrants to America, have come from Italy. The Italians are great travelers, some crossing and recrossing the ocean many times, according to fluctuations of business.

Italians.

| Entered U. S. (North) | Entered U. S. (South) | Left U. S. (North) | Left U. S. (South) | |
|---|---|---|---|---|
| 24,700 | 110,547 | 19,507 | 147,828 | Year ending June 30, 1908 |
| 34,116 | 212,857 | 24,773 | 101,063 | Year ending June 30, 1909 |
| 35,192 | 215,445 | 22,591 | 63,554 | Year ending June 30, 1910 |
| 37,253 | 176,107 | 24,065 | 93,297 | Year ending June 30, 1911 |
| 34,243 | 155,680 | 25,857 | 139,421 | Year ending June 30, 1912 |
| 165,504 | 870,636 | 116,793 | 545,163 | Totals for five years. |

It is quite likely that the decrease in immigration and increase in emigration in the last two years is due partly to the war.

---

Patriotism and Migration. There has long been a prejudice against the southern tier of European countries or it may be that the general prejudice against the foreigner and the mutual prejudices among various kinds of foreigners have been thus directed on account of the influence of numbers and the making good of several generations of foreigners from more northern European countries to America. Recent events in China and in what, in our school days was called and looked like Turkey in Europe, show a surprising resemblance to the initiative, courage, patriotism and military genius displayed in this country in the third quarter of the eighteenth century. Even more plainly than in the case of the Italians, does the patriotic element appear in emigration statistics.

Entered U. S.

| Bulgarians | Roumanians | Greeks | Turks | Year Ending June |
|---|---|---|---|---|
| 18,246 | 9,629 | 28,808 | 2,327 | 1908 |
| 7,623 | 9,154 | 22,453 | 925 | 1909 |
| 16,374 | 14,954 | 41,172 | 1,422 | 1910 |
| 10,924 | 5,823 | 38,954 | 1,005 | 1911 |
| 12,698 | 9,436 | 34,243 | 1,430 | 1912 |
| 3,256 | 2,464 | 21,535 | 494 | 1912 July-Sept. |

(Immigration not obtainable for October, 1912.)

Left U. S.

| | | | | |
|---|---|---|---|---|
| 5,965 | 5,264 | 6,763 | 1,276 | 1908 |
| 3,626 | 2,102 | 9,128 | 1,150 | 1909 |
| 4,105 | 2,647 | 10,624 | 1,564 | 1910 |
| 8,700 | 7,108 | 14,419 | 2,290 | 1911 |
| 12,554 | 8,080 | 25,857 | 2,076 | 1912 |
| 1,614 | 1,238 | 4,952 | 587 | 1912 July-Sept. |
| 1,057 | 405 | 1,764 | 277 | 1912 Oct. |

The above figures would be even more significant if the sexes were distinguished. For example, about 800 Greeks left St. Louis in a body, this year to engage in the war and we understand that the emigration has been largely of males while women and children have figured disproportionately in the immigration.

---

Physicians Wanted. The Pacific Medical Bureau, 1065 Sutter St., San Francisco, announces a number of desirable locations in California.

---

Autopsy Pledges. The Associated Physicians of Long Island have adopted a resolution aiming to secure pledges from its members that they will require their heirs to allow post mortem examinations.

---

Consumption of Cigarettes in the U. S. The *Burning Bush* renders statistics startling by figuring a death's head in the smoke from a cigarette, along with the statement that 11,221,624,084 cigarettes were smoked in the last fiscal year. However, when we divide this by 100 million, the approximate population, the per capita consumption of 112 cigarettes does not look so imposing. Allowing for the fact that about a quarter of any community consists of grown men, the consumption would be about 500, per capita. This is equivalent to a moderate use of tobacco in other forms—for a single month—say 10 mild cigars a week or a half an ounce a day of pipe tobacco. Right here is a good place to waste an editorial heading: *Don't let the Dividend alarm you; It's the Quotient that counts.*

---

Contaminated Artesian Wells. South Bethlehem, Pa., is said to have had the first system of water works in the U. S., deriving the supply from springs in 1741. This supply has been in constant use till recently, when $50,000 were spent for an Artesian supply. Almost as soon as the new supply was used, the water was found to contain colon bacilli and the town has temporarily returned to the old supply, with precautions, pending the establishment of a filtration plant for the Artesion supply. This unfortunate occurrence opens up many practical problems in engineering and leads us to inquire whether the water obtained by various hotels and factories from deep wells, not deserving the term Artesian, in our own territory, has been subjected to thorough tests.

---

Sanitary Reforms. In St. Louis the Council of Civic Organizations, comprising eighteen of the leading organizations which are working for civic improvement, with a membership of 8,000 proposes:

Wrapping of bakery products in clean paper.

Sanitary care of tenements and running water on each floor of such buildings.

Abolition of the 20,000 vaults in St. Louis within five years.

To regulate the city's milk supply to insure absolutely clean and pure milk.

Proper heating and ventilation of street-cars.

---

Number of Words. We have been criticized for questioning so good an authority as Max Müller in regard to the extent of one's vocabulary. It is, in the first place, impossible to make an accurate count of words. Our Webster is said to contain about 114,000 words. On an average count of several pages, we find the number to be about 85,000. The discrepancy is due largely to counting the same words, used as a noun and as a verb, as one, and to ignoring inflexions. Many of the words are compound and, in this case, it is difficult to establish a rule. The dictionary apparently lists all compound words whose meaning is not readily suggested by the components. On averaging counts of several pages, we found that we could understand fairly well, the significance of about 60 per cent of the words. About a third of this number, we would be likely to use, representing a vocabulary of somewhere between 15,000 and 25,000 words; and three times as many understandable. Of course, if one took time to puzzle over obsolete and unusual words, which he did not know were in use at all, counted verbs and nouns of the same form as different words, and otherwise followed the more liberal count of the dictionary, one might claim to know 50,000 to 75,000 words. On the other hand, if one counted defender, defendant, defensive, defensible, defense, etc., as variants of defend, the vocabulary would be reduced to about a fifth and in this sense, Max Müller may be right in stating that a man with a university education would use only 3,000 or 4,000 words, especially when we recall that he referred directly to the German language which, even for technical terms, relies mainly on home-made compounds. But, as words are ordinarily counted, most fairly well educated persons have a usable vocabulary of at least ten thousand words. Indeed, the little vest pocket dictionaries for travelers contain as many, which are needed for ordinary purposes. Including technicalities, the average physician probably knows at least 50,000 words.

---

Members of Medical Reserve Corps to Be Full Surgeons. On account of the unwillingness of prominent physicians to accept the rank of Assistant Surgeon, the Bureau of Medicine and Surgery will ask Congress to designate members of this corps as full Surgeons, there being but one rank in the corps. This is really a sensible proposition though, at first, it reminded us of early companions who would not play unless they could be captain.

---

The Mowatt Memorial Hospital was opened at Kingston, Dec. 12.

---

Disclaimer of Notoriety. The Ferment Co., of 124 W. 31st St., N. Y., disclaims the yellow press reports of the activity of its products in diabetes and other diseases and wishes the profession to judge them in accordance with conservative, scientific articles in the medical press. The sensational articles were published without authority by the firm, based on information given to physicians in response to what was considered a legitimate professional inquiry.

---

Corning Water Supply. Many Corning physicians have protested to the Board of Health against using the present supply, which is highly charged with lime and which is polluted. They object to drinking even sterilized sewage.

SYSTEMATIC COLLABORATION REGARDING SANITARY CONDITIONS has been enjoined upon local health officers in Ontario, by Dr. J. W. S. McCullough, Provincial Health Officer.

---

MEDICAL SOCIETY OF THE STATE OF NEW YORK. Committe on prize essay. The committee in charge of the Merritt H. Cash, $100.00, and the Lucian Howe, $100.00, Prize Fund of the Medi-Cal Society of the State of New York, offer the suggestive, but not arbitrary, subjects upon which the competitors may write their essays:—

1. Diagnosis and treatment of syphilis of the central nervous system.

2. The present status of serum therapy.

3. Latest research relative to cancer.

4. The order and sequence of vascular and cardiac disease.

5. The function of the State in limiting the increase of imbeciles and degenerates.

6. Surgery in functional and organic disorders of the stomach.

The essays must be in the hands of the Chairman of the Committee, Dr. Albert VanderVeer, 28 Eagle Street, Albany, N. Y., not later than April 1, 1913.

Dr. John F. W. Whitbeck, 781 Park Ave., Rochester, N. Y.
Dr. Edward D. Fisher, 46 East 52d St., New York.
Dr. Albert VanderVeer, 28 Eagle St., Albany, N. Y.
Committee.

---

SCHEDULE OF FEES. The Chemung Co. supervisors have established the following: $10 for each examiner in an ordinary autopsy; $20 in a homicide case; $15 for examinations in lunacy.

---

FAITH HEALER DISCHARGED. The Medical Society of the County of Erie has recently lost its case against a faith healer in Buffalo. The evidence depended, as is usually the case in such prosecutions, upon a detective and such evidence is always discounted to some degree. The jury held that the defendant had the right to heal according to his religious views while his attorney made the stock accusation that the medical profession was actuated by mercenary motives to stop competition. Hic populus vult decsepi; decipiatur in nomine diaboli.

The contract for the FRANCES ELLICOTT AUSTIN MATERNITY HOSPITAL, at Albany, has been let at about $175,000.

---

EXAMINATIONS FOR U. S. ARMY MEDICAL CORPS, will be held at points to be named in accordance with applications received, on Jan. 20. There are 35 first lieutenancies in the medical corps to be filled. The only requirement that is not obvious is a year's interne service. Applications should be made promptly to the Surgeon-General, U. S. Army, at Washington. Here is an opening for almost one per cent. of the total graduates of 1911. It is probably not an exaggeration to claim that 10 per cent of all medical graduates may obtain excellent public and institutional positions, at salaries exceeding the average professional income, almost immediately after completing their interne service.

---

COUNTY HOSPITALS FOR TUBERCULOSIS. The Steuben County supervisors voted against the purchase of 20 acres near Corning, at $1,000, by 27 to 10. The Livingston County supervisors considered a petition on Nov. 21 and appointed a committee to investigate and report at a future meeting.

---

MEDICAL MISSIONARY OPENINGS. Mr. Wilbert B. Smith, Candidate Secretary, Student Volunteer Movement, 125 E. 27th St., N. Y. City announces the following: Woman physician for the hospital and dispensary for Women and Children at Jhansi, India; Woman physician for north-eastern Korea, with headquarters at Jen San; five trained nurses for hospitals in Turkey, India and Ceylon; two years interneship, Good Samaritan Hospital, Guanaquato, Mex. It should be understood that while these positions require considerable ability and a sincere devotion to the missionary movement, they are adequately paid.

---

HYPERPAEDIOPHILIA. (We think that will hold some of the men who like big words.) A Cleveland woman has recently been arrested and placed in the observation ward of the City Hospital, for the deliberate attempt to keep her three children babies. Aged 2, 3, and 4, they were unable to walk or talk and only the oldest could creep, as she had kept them in cradles to prevent their development. It is said that they were even stunted in growth. Most parents feel a tinge of sadness at the growth of their children from stage to stage and we have read a story of this sentiment exaggerated as in the real case mentioned—which is, fortunately a rare one.

---

ILLEGAL USE OF MAILS. One of the largest raids made by the P. O. Dept. occurred late in November. Inspectors had been watching various quacks, druggists and medical mail order houses on account of the use of the mails for objectionable medical and surgical devices. The raid covered 72 cities in 22 states and involved 175 individual offenders.

---

TUBERCULOSIS AND WATER SUPPLY. The Westchester supervisors have been denied permission to establish a tuberculosis hospital at Yorktown, on account of possible contamination of the N. Y. City water supply.

---

H. C. OF L. AND BUTTER. The United States has begun suit against the Elgin Board of Trade and the Am. Assn. of Creamery Butter Manufacturers as "in restraint of trade."

---

SAFE AND SANE FOOTBALL. Only 10 deaths and 36 serious accidents were reported in the U. S. for 1912, up to Dec. 1.

---

THE INTERNATIONAL JOINT COMMISSION met in Buffalo, Dec. 17, to consider the pollution of boundary waters between the U. S. and Canada.

---

OBESITY. Frank Chambers, of North Pelham Tp, Ont., was found dead in bed, Dec. 13, aged 43. He weighed 540 pounds and was considered the largest man in Canada.

---

U. S. PUBLIC HEALTH SERVICE EXAMINATIONS, for admission to the grade of Assistant Surgeon, will be held in Washington, Jan. 13. Application should be made promptly to the Surgeon General.

RETIRED AND NON-PRACTICING PHYSICIANS. The Medical Directory of N. Y., N. J., and Ct., has a special section under this title. Frequently the death is announced of some physician who ought to be thus listed but who is not. We also note in the active lists of Buffalo and other cities, a considerable number of names of physicians engaged in various occupations, among them the very laudable one of helpmate to another physician.

---

EARLY IMMIGRATION. It is stated that 20,000 English settled in New England between 1620 and 1640.

---

THE HOUSEWIVES' LEAGUE has succeeded in restoring the price of milk to 7 cents in Buffalo and in supplying good eggs at 21 cents a dozen when eggs of corresponding quality have been selling at 35 cents or more. It insists that, when it buys in wholesale quantities it shall have wholesale rates. It is carrying on an active campaign of education along both hygienic and economic lines and is perfecting its organization and increasing its membership. It is urged that the women of physicians' families should take an active part in this movement. Item by item, the league proposes to attack the problem of the increased cost of living, which is mainly the increased cost of foods, but in no narrow spirit. The recent government suit against the Butter Trust is said by local commission merchants not to be likely to affect the local market, as it is claimed that prices here have not been dictated by the trust but by the law of supply and demand, however, it is probable that, by direct buying from dairies, the league will considerably reduce the price of butter within a few weeks.

---

THE COLLEGE OF MEDICINE OF SYRACUSE UNIVERSITY announces the laying of the corner stone of the new dispensary building on Dec. 14. Chancellor James R. Day officiated. Wm. S. Thayer, M.D., LL.D., of John Hopkins, August S. Downing, First Assistant Commissioner of Education of New York State, and Alan C. Fobes in behalf of the Syracuse Free Dispensary Association gave addresses. The building will be completed by the beginning of the next college term.

---

MEDICAL STUDENTS STRIKE. It is reported that nine seniors from a Milwaukee medical school have left for a St. Louis institution and that about 250 students have ceased attending, on account of a reduction in rating by the A. M. A.

